# Associations between Vaginal Pathogenic Community and Bacterial Vaginosis in Chinese Reproductive-Age Women

**DOI:** 10.1371/journal.pone.0076589

**Published:** 2013-10-04

**Authors:** Zongxin Ling, Xia Liu, Yueqiu Luo, Xiaoxing Wu, Li Yuan, Xiaojuan Tong, Lanjuan Li, Charlie Xiang

**Affiliations:** 1 State Key Laboratory for Diagnosis and Treatment of Infectious Diseases, The First Affiliated Hospital, School of Medicine, Zhejiang University, Hangzhou, Zhejiang, China; 2 Collaborative Innovation Center for Diagnosis and Treatment of Infectious Diseases, Hangzhou, Zhejiang, China; 3 Department of Intensive Care Unit, The First Affiliated Hospital, School of Medicine, Zhejiang University, Hangzhou, Zhejiang, China; 4 J. Craig Venter Institute, Rockville, Maryland, United States of America; University of Iowa Carver College of Medicine, United States of America

## Abstract

**Background:**

Bacterial vaginosis (BV) is one of the most common urogenital infections among women of reproductive age that represents shifts in microbiota from *Lactobacillus* spp. to diverse anaerobes. The aim of our study was to evalute the diagnostic values of *Gardnerella*, *Atopobium*, *Eggerthella*, *Megasphaera* typeI, *Leptotrichia*/*Sneathia* and *Prevotella*, defined as a vaginal pathogenic community for BV and their associations with vaginal pH and Nugent scores.

**Methods and Findings:**

We investigated the vaginal pathogenic bacteria and *Lactobacillus* spp. with species-specific real-time quantitative PCR (qPCR) in 50 BV-positive and 50 BV-negative Chinese women of reproductive age. Relative to BV-negative subjects, a siginificant decline in *Lactobacillus* and an obvious increase in bacteria in the vaginal pathogenic community were observed in BV-postive subjects (P<0.05). With the exception of *Megasphaera* typeI, other vaginal pathogenic bacteria were highly predictable for BV with a better sensitivity and specificity. The vaginal pathogenic community was positively associated with vaginal pH and Nugent scores, while *Lactobacillus* spp., such as *L. iners* and *L. crispatus* was negatively associated with them (P<0.05).

**Conclusions:**

Our data implied that the prevalance of vaginal pathogenic bacteria as well as the depletion of *Lactobacillus* was highly accurate for BV diagnosis. Vaginal microbiota shifts, especially the overgrowth of the vaginal pathogenic community, showed well diagnostic values in predicting BV. Postive correlations between those vaginal pathogenic bacteria and vaginal pH, Nugent score indicated the vaginal pathogenic community rather than a single vaginal microorganism, was participated in the onset of BV directly.

## Introduction

Bacterial vaginosis (BV) is the most prevalent lower genital tract infection in women of reproductive age worldwide [1], [2]. Previous research has shown that BV is an ecological disorder of the vaginal microbiota that affects millions of women annually [3]–[6] and is associated with numerous adverse health outcomes, including preterm birth and the acquisition of sexually transmitted infections [7], [8]. BV can be characterized microbiologically by replacement of the lactobacilli-predominant vaginal microbiota by vaginal pathogenic bacteria. The dramatic shifts in vaginal microbiota from a healthy, H_2_O_2_- and lactic acid-producing lactobacilli-dominated microbiota to a complex multispecies microbiota that contributes to pH elevation and sialidase and amine production, and eventually leads to the observed signs and symptoms of BV [9], [10]. The advent of culture-independent molecular approaches based on high-throughput sequencing techniques has furthered our understanding of the vaginal microbiota by identifying taxa that have not been cultured [4]–[6], [9]. Our previous studies have demonstrated that a group of microorganisms are present concurrently in high concentrations in the vaginas of women with BV, which are reduced significantly after successful treatment [4], [6]. The collective BV-associated microorganisms are defined as a vaginal pathogenic community, including *Gardnerella*, *Atopobium*, *Eggerthella*, *Megasphaera* typeI, *Leptotrichia*/*Sneathia* and *Prevotella* [4]–[6]. In fact, these bacteria in the vaginal pathogenic community mentioned above are not isolated from one another; instead, each microorganism can interact with others that are involved in the development of BV. Thus, clarification of the relationship between vaginal pathogenic bacteria and the development of BV and the associations between vaginal pathogenic bacteria and BV-associated changes, such as vaginal pH and Nugent scores, are needed.

With a specific quantitative real-time PCR (qPCR) assay, our present study determined the relationship between the relative abundance of vaginal pathogenic bacteria and BV in Chinese women of reproductive age, which will demonstrate the diagnostic value of BV and the associations with vaginal pH and Nugent scores. Our study will shed light on the etiology of BV based on a new concept involving the vaginal pathogenic community, rather than a single vaginal microorganism, which participates in the process of BV.

## Materials and Methods

### Subjects

One hundred women with regular menstrual cycles (24–35 days), 19–51 years of age, including 50 BV-positive women (BV group; 33.3±9.1 years of age) and 50 healthy control women (CN group; 32.0±8.1 years of age), who presented to the Department of Obstetrics and Gynecology of the First Affiliated Hospital, School of Medicine, Zhejiang University for routine gynecology examination between October 2008 and May 2009, were recruited for this study [4]. Informed written consent was obtained from all participants prior to enrollment. The study protocol was approved by the Ethics Committee of the First Affiliated Hospital, School of Medicine, Zhejiang University (Zhejiang, China). Individuals who participated in this study were examined by two gynecologists. BV status was assessed using the Amsel clinical criteria for all subjects [11], and confirmed using Gram staining criteria (Nugent scores) [12]. Only participants with Nugent scores ≥7 were selected as BV for the following analysis. The inclusion and exclusion criteria were described in our previous study [4]. Participants without any abnormal changes were defined as the BV-negative group.

### Sample Collection and Preparation

When women underwent genital examinations, two samples were obtained near the mid-vagina using a sterile swab, packaged, and placed in ice packs. The first swab was rolled onto a slide for Gram staining; the second vaginal swab was used for bacterial genomic DNA extraction. The vaginal swabs for bacterial genomic DNA extraction were transferred to the laboratory immediately in an ice box and stored at −80°C after preparation within 15 min for further analysis.

### Total bacterial genomic DNA extraction

The bacterial cells retrieved on swabs were submerged in 1 ml of sterile normal saline (prepared with RNase free H_2_O [pH 7.0]) and vigorously agitated to dislodge cells. The cells were pelleted by centrifugation (Thermo Electron Corporation, Boston, MA, USA) at full speed (≥10,000 g) for 10 min, washed by re-suspending cells in sterile normal saline and centrifuged at full speed for 5 min. Then bacterial DNA was extracted from the vaginal swabs using a QIAamp DNA Mini Kit (QIAGEN, Hilden, Germany) according to the manufacturer's instructions with minor modifications as previous described [4]–[6], [13]–[15]. Bacterial genomic DNA was eluted with elution buffer and stored at −20°C for further analysis.

### Real-time qPCR for vaginal pathogenic bacteria

To estimate the accurate loads of pathogenic bacteria in vaginal samples, 16S ribosomal RNA (rRNA) gene-targeted qPCR was performed with a Power SYBR Green PCR Master Mix (Takara, Dalian, China) on an ABI 7900 Real-time PCR instrument according to the manufacturer's instructions (Applied Biosystems, Foster City, CA, USA). Species-specific primer sets were chosen to quantify total bacteria, *Lactobacillus genus*, *L. iners*, *L. crispatus*, *L. jensenii*, *Gardnerella vaginalis*, *Atopobium vaginae*, *Eggerthella* spp., *Megasphaera* typeIspp., *Leptotrichia*/*Sneathia* spp., and *Prevotella* spp. [4], [5]. For each primer set, a constructed plasmid was chosen to create a 10-log-fold standard curve for direct quantification of all samples. With the exception of total domain *Bacteria* and *Lactobacillus genus*, all standard curve genes were amplified from the vaginal samples, plasmids were constructed, sequenced and the source of target organisms was confirmed by BLAST in GenBank. For total domain *Bacteria* and *Lactobacillus genus*, *E. coli* ATCC 25922 and *L. casei* ATCC 27139 were used to create the plasmid standards, respectively. For each plasmid standard, the product was cloned into pMD18-T vector using the Simple TA Cloning Kit (Takara, Dalian, China) following the manufacturer's procedure. Purified insert-containing plasmids were quantified using a NanoDrop ND-1000 spectrophotometer (Thermo Electron Corporation). Taking into account the size of the product insert, the number of target gene copies was calculated from the mass of DNA. Ten-fold serial dilutions, ranging from 1×10^9^ to 1 gene copy, were included on each 96-well plate. The extracted DNA was subjected to a human β-Globin PCR to ensure that amplifiable DNA was successfully extracted from the sample and to monitor for PCR inhibitors with the same protocol listed for bacterial PCR [16]. Each qPCR contained 12.5 µL of 2× Takara Perfect Real Time master mix, 10.9 µL of water, 0.3 µL of a 10 µM F/R primer mix, and 1 µL of extracted bacterial genomic DNA. The cycling conditions were as follows: 95°C for 3 min; and 40 repeats of 94°C for 30 s, 30 s annealing at different temperatures, and 72°C for 30 s. At each cycle, accumulation of PCR products was detected by monitoring the increase in fluorescence of the reporter dye, dsDNA-binding SYBR Green. Following amplification, melting temperature analysis of PCR products was performed to determine the specificity of the PCR. Melting curves were obtained from 55–90°C, with continuous fluorescence measurements obtained at every 1°C increase in temperature. Data analysis was conducted with Sequence Detection Software (version 1.6.3; Applied Biosystems, Foster City, CA, USA). All reactions were carried out in triplicate and a non-template control was performed in every analysis. In addition, the abundance of each group relative to total domain *Bacteria* gene copy number was calculated for each replicate, and the mean, standard deviation, and statistical significance were determined.

### Statistical analysis

Comparisons of pathogenic bacteria loads between women with and without BV were calculated with the Mann-Whitney *U*-test. Correlation coefficients and significant P values between vaginal pH, Nugent scores, and vaginal pathogenic microorganisms were also calculated. Receiver-operator characteristic (ROC) analyses were also performed with vaginal pathogenic bacteria plotted against BV. The area under ROC curve (AUC) was estimated to assess the predictive power. Diagnostic accuracy was categorized as failed (ROC AUC ≤0.6), poor (0.6< ROC AUC ≤0.7), fair (0.7<ROC AUC ≤0.8), good (0.8<ROC AUC ≤0.9) or excellent (0.9<ROC AUC≤1.0). The sensitivity, specificity, negative and positive predictive values for the detection of BV were determined for the vaginal pathogenic bacteria and the genus of *Lactobacillus*. The diagnostic accuracy of the vaginal pathogenic bacteria and the best cut-off points were determined with ROC curves. SPSS16.0 software (SPSS Inc., Chicago, IL, USA) was used for all statistical analyses. Two-tailed P values with composite results at a P<0.05 were considered statistically significant.

## Results

### Vaginal pathogenic bacteria associated with BV

**Figure 1:**
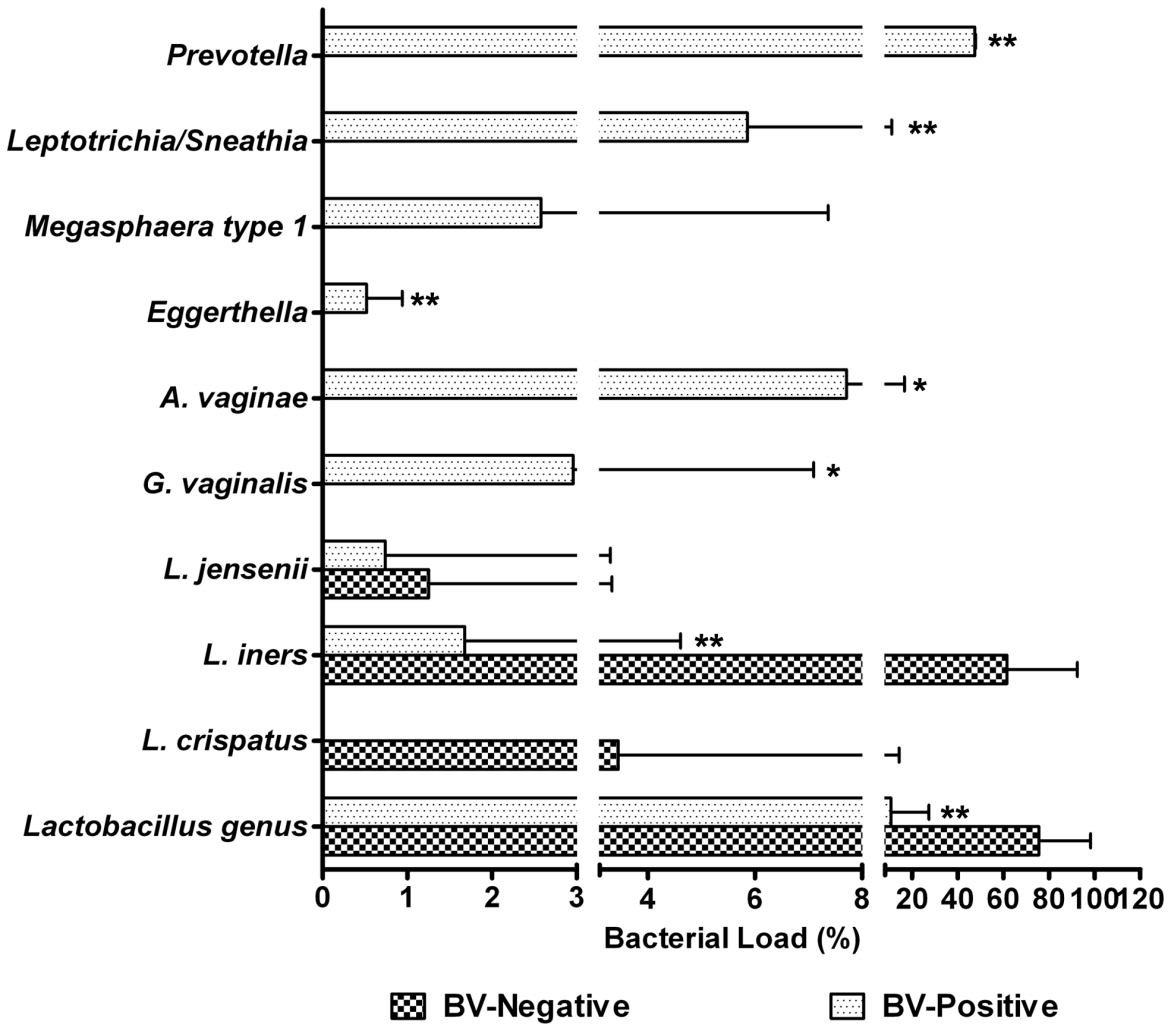
*Lactobacillus* spp. and the vaginal pathogenic bacteria quantified by species-specific quantitative PCR (qPCR). The relative abundance of the vaginal pathogenic bacteria and *Lactobacillus* were compared to the copy number of total bacteria. The relative abundance of *L. crispatus*, *L. iners* and *L. jensenii* was compared to the copy number of *Lactobacillus*. The Mann-Whitney *U*-test was used to evaluate statistical difference between the BV-positive and BV-negative groups. * indicates P<0.05; ** indicates P<0.01.

Figure 1 gives an overview of the results obtained with real-time qPCR for three vaginal *Lactobacillus* spp. and six vaginal pathogenic bacteria. Generally, the total bacteria in the vagina with or without BV were nearly the same in one mid-vaginal swab (P>0.05), which provides good comparability for these bacteria, even in the absolute abundances in the vagina. Consistent with previous findings, we showed that there was a profound shift of lactobacilli present in the vagina when comparing populations associated with healthy and diseased conditions. In the BV-positive subjects, lactobacilli was reduced significantly, and even disappeared in some samples (P<0.05). One of the *Lactobacillus* spp., *L*. *iners*, was a major component of the vaginal microbiota in healthy women and decreased markedly in BV-positive subjects (P<0.05), while two other predominant species (*L. crispatus* and *L. jensenii*) were also reduced in BV-positive subjects, but did not reach statistical significance (P>0.05). Of vaginal pathogenic bacteria, *Gardnerella*, *Atopobium*, *Megasphaera* typeI, *Eggerthella*, *Leptotrichia*/*Sneathia*, and *Prevotella* were more common and present in higher copy numbers in the BV-positive group. With the exception of *Megasphaera* typeI, the other vaginal pathogenic bacteria mentioned above were increased markedly in BV-positive subjects (P<0.05). Although the changing patterns were not always the same for all subjects, our quantitative study involving the vaginal pathogenic bacteria demonstrated one common finding, specifically, an increased number of these bacteria were found during the advent of BV. Relative to BV-negative subjects, the relative abundance or copy number of these bacteria combined with each other was significantly associated with BV and could be potentially used as a molecular marker of a microbiota shift in the vagina and as a target for the diagnosis of BV.

### Vaginal pathogenic bacteria as potential predictors of BV

**Figure 2:**
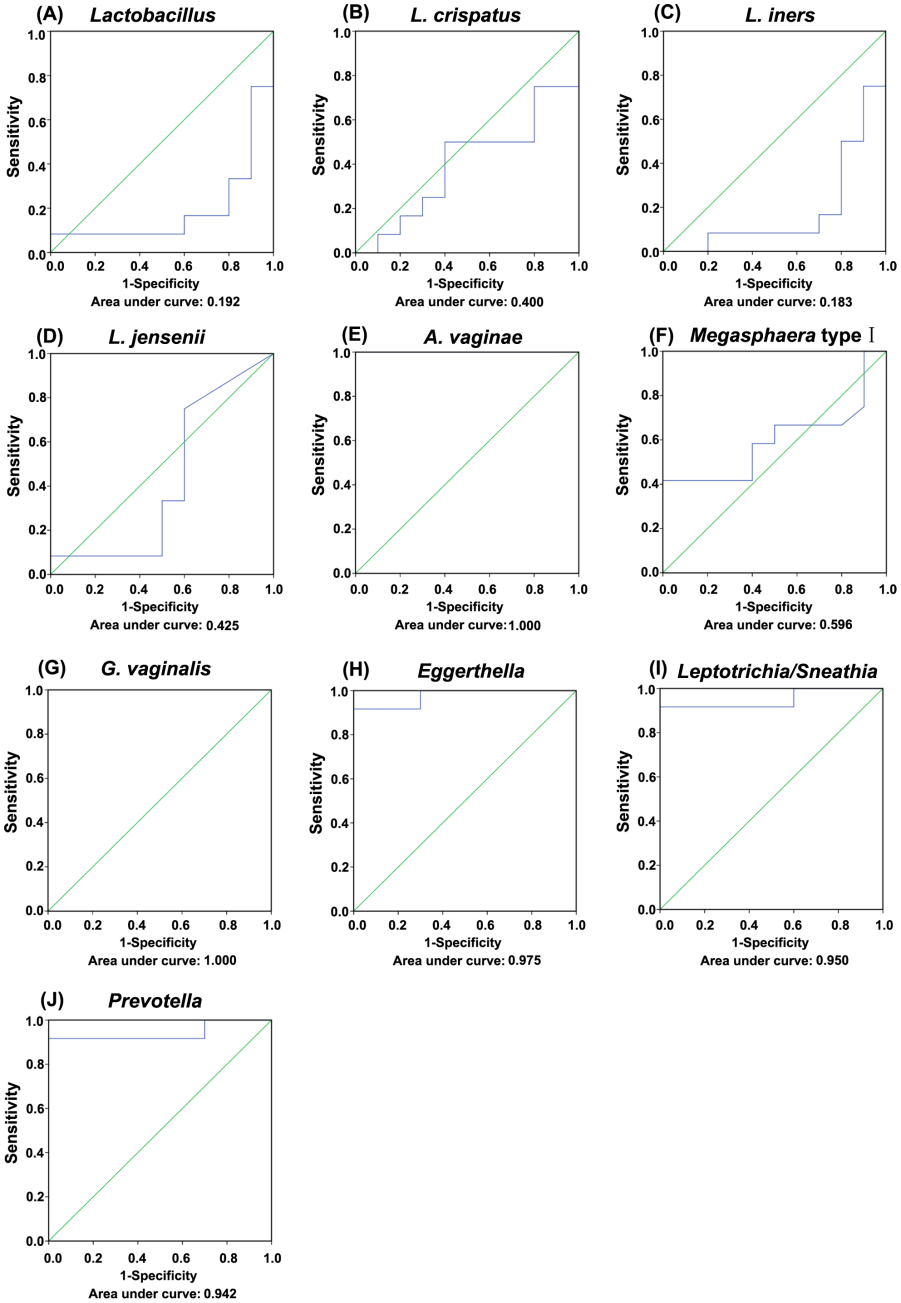
Receiver operating characteristic (ROC) curves for bacterial molecular counts used to predict bacterial vaginosis (BV). The closer the area under the curve (AUC) is to 1.0, the better the bacterial counts predict BV. *A. vaginae* (E) and *G. vaginalis* (G) counts have the best predictive power for BV, while *Eggerthella* (H), *Leptotrichia*/*Sneathia* (I) and *Prevotella* (J) have the better predictive power for BV. (A) *Lactobacillus* ROC curve; (B) *L. crispatus* ROC curve; (C) *L. iners* ROC curve; (D) *L. jensenii* ROC curve; (F) *Megasphaera* typeIROC curve.

ROC analysis was performed to evaluate the predictive power of vaginal pathogenic bacteria for BV. When a comparison was made between *Lactobacillus* spp. and BV, the AUC was less than 0.500. When a comparison was made between *A. vaginae*, *G. vaginalis* and BV, the AUC was 1.000 (P<0.01), which showed the best diagnostic accuracy. When a comparison was made between *Eggerthella* and BV, the AUC was 0.975 (P<0.01). When a comparison was made between *Leptotrichia/Sneathia* and BV, the AUC was 0.950 (P<0.01), while in a comparison between *Prevotella* and BV, the AUC was 0.942 (P<0.01; Figure 2). Table 1 demonstrated the sensitivity, specificity, negative and positive predictive values of the vaginal pathogenic bacteria with the best cut off points (the abundance of vaginal bacteria relative to total Bacteria gene copy number). *G. vaginalis*, *Prevotella* and *A. vaginae* showed the best sensitivity for BV (100%, 100% and 98%), while *Eggerthella* and *Leptotrichia/Sneathia* showed the best specificity (100% and 98%). Our data indicated that these vaginal pathogenic bacteria such as *G. vaginalis*, *Prevotella*, *A. vaginae*, *Eggerthella* and *Leptotrichia/Sneathia* were highly predictable for BV, with excellent diagnostic accuracy.

**Table 1:** Sensitivity, specificity and predictive values of the vaginal pathogenic bacteria and Lactobacillus for the prediction of BV.

Bacteria	Sensitivity	Specificity	Positive predictive value (%)	Negative predictive value (%)
*Lactobacillus genus* (8.0260%)^1^	12	98	2.2	10.9
*G. vaginalis* (0.00004%)^1^	100	68	100	75.8
*A. vaginae* (0.00056%)^1^	98	68	97.1	75.4
*Eggerthella* (0.00460%)^1^	80	100	83.3	100
*Megasphaera* typeI (0.00028%)^1^	38	90	79.2	59.2
*Leptotrichia/Sneathia* (0.22304%)^1^	78	98	97.5	81.7
*Prevotella* (0.00338%)^1^	100	78	82	100

1 Cut-off level.

### Associations of vaginal pathogenic bacteria with vaginal pH

**Figure 3:**
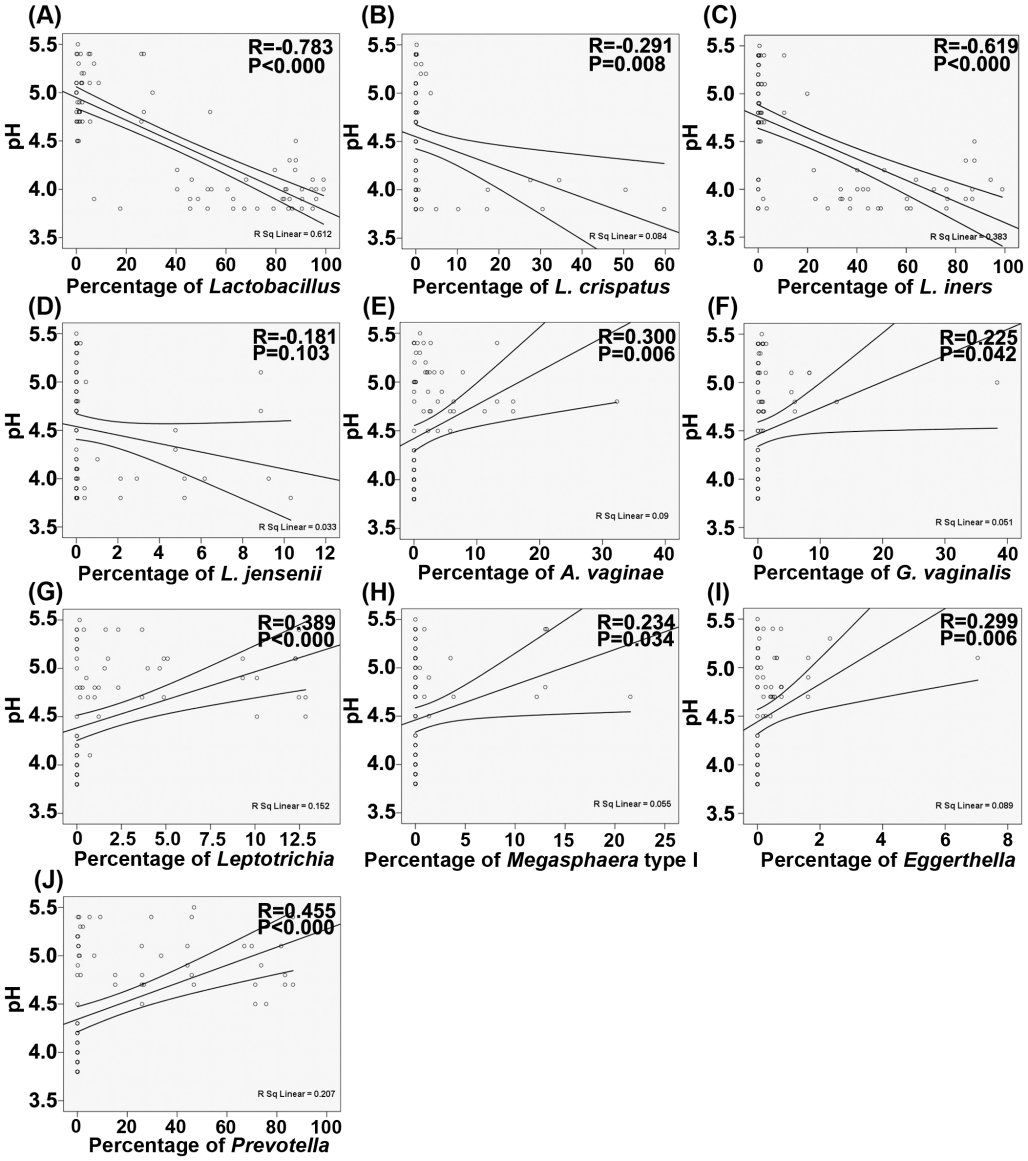
Correlations between *Lactobacillus* spp., the vaginal pathogenic bacteria and vaginal pH. (A) *Lactobacillus*. (B) *L. crispatus*. (C) *L. iners*. (D) *L. jensenii*. (E) *Atopobium vaginae*. (F) *Garnerella vaginalis*. (G) *Leptotrichia*/*Sneathia*. (H) *Megasphaera* typeI. (I) *Eggerthella*. (J) *Prevotella*. The Pearson correlation (R) and probability (P) were used to evaluate statistical importance.

Vaginal pH was a sensitive and simple maker for monitoring the vaginal microenvironment, which was changed according to a shift in the vaginal microbiota. Figure 3 demonstrates that there were negative associations between *Lactobacillus*, *L. crispatus*, *L. iners*, and vaginal pH that was identified by computing the Pearson correlation coefficients (P<0.05), while there was no relationship between other *Lactobacillus* spp., such as *L. jensenii* and vaginal pH (P>0.05). In addition, there were positive relationships between vaginal pathogenic bacteria and vaginal pH (P<0.05). The present data indicate that vaginal pH is significantly correlated with vaginal pathogenic bacteria.

### Associations of vaginal pathogenic bacteria with Nugent scores

**Figure 4:**
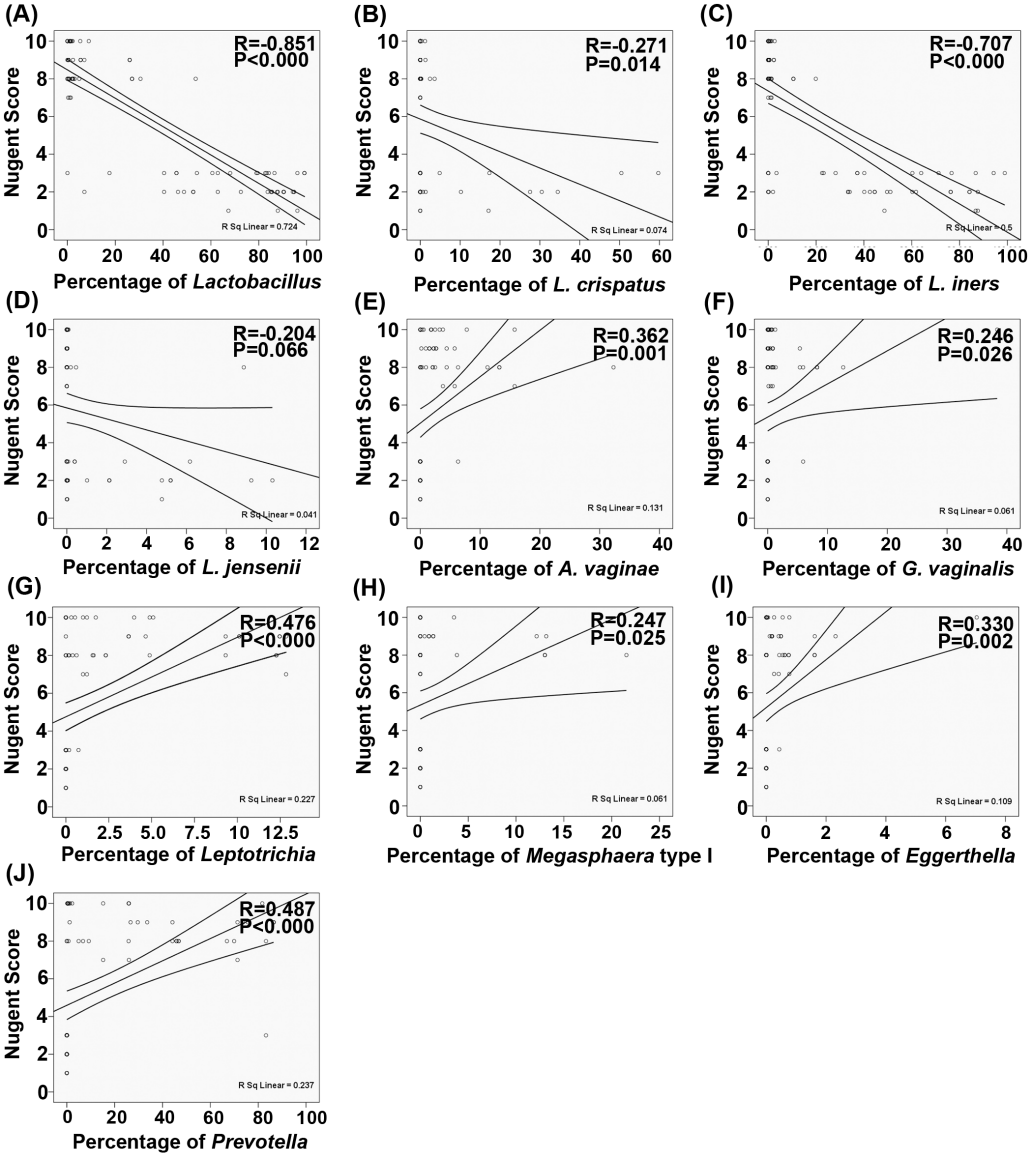
Correlations between *Lactobacillus* spp., the vaginal pathogenic bacteria and Nugent scores. (A) *Lactobacillus*. (B) *L. crispatus*. (C) *L. iners*. (D) *L. jensenii*. (E) *Atopobium vaginae*. (F) *Garnerella vaginalis*. (G) *Leptotrichia*/*Sneathia*. (H) *Megasphaera* typeI. (I) *Eggerthella*. (J) *Prevotella*. The Pearson correlation (R) and probability (P) were used to evaluate statistical importance.

The Nugent score was a diagnostic factor commonly used to identify women with BV. Based on weighted counts of different cellular morphotypes, Nugent (microbiological) scores for BV were categorized as normal vaginal microbiota (score, 0-3), intermediate microbiota (score, 4–6), or BV (score, 7–10). In the present study, there were negative associations between *Lactobacillus*, *L. crispatus*, *L. iners* and Nugent scores that were identified by computing the Pearson correlation coefficients (P<0.05). Most of these vaginal pathogenic bacteria were gram-negative bacteria, which corresponded to higher Nugent scores. The present study demonstrated that there were positive correlations between vaginal pathogenic bacteria and Nugent scores (P<0.05; Figure 4).

## Discussion

As one of the complex ecological niches, the vagina harbors numerous microorganisms that play crucial roles in preventing a number of urogenital diseases. To date, nearly 400 species-level phylotypes have been discovered in vaginal habitats [9]. Our knowledge of vaginal microbial diversity has expanded enormously through the use of culture-independent approaches based on the analysis of 16S rRNA gene sequences. It is generally accepted that a shift in microbial composition is an important step in the progression of urogenital diseases. Unlike any other anatomic site on the human body, most vaginal communities are dominated by one or more species of *Lactobacillus* that constitute >50% of total bacteria in a healthy vaginal ecosystem. The onset of BV is marked by a decline in the *Lactobacillus* spp. and other facultative or anaerobic species as the vaginal microbial ecosystem changes from eubiosis to dysbiosis [8]. As BV progresses, the healthy vaginal microbiota is gradually replaced by vaginal pathogenic bacteria, which will disturb the balance of vaginal microecology and is significantly associated with BV. We showed that a decrease in *Lactobacillus* was accompanied by an increase in the vaginal pathogenic bacteria, such as *Gardnerella*, *Atopobium*, *Eggerthella*, *Megasphaera* typeI, *Leptotrichia*/*Sneathia* and *Prevotella*. Consistent with previous studies on the predominant bacteria of the vaginal microbiota, our real-time qPCR data also detected a significant decrease in the relative abundance of *Lactobacillus* [3]. Members of the genus *Lactobacillus* are commonly identified as the hallmark of a normal or healthy vagina [17]. Although 13 different *Lactobacillus* spp. have been detected in healthy vaginas [18], 3 predominant *Lactobacillus* spp. (*L. crispatus*, *L. iners* and *L. jensenii*) account for the largest proportion of normal vaginal microbiota. In the present study, *L. iners* was the most prevalent *Lactobacillus* spp., followed by *L. crispatus* and *L. jensenii*. Pavlova *et al*. (2002) have also reported that *L. crispatus* is the predominant species in most populations, which may be associated with different genetic factors [19]. The increase in the relative abundance of *A. vaginae*, *G. vaginalis*, *Eggerthella*, *Leptotrichia/Sneathia* and *Prevotella* had good diagnostic value for BV, and might be independent markers for the diagnosis of BV. Two familiar bacteria, *A. vaginae* (DNA level, ≥10^8^ copies/mL) and *G. vaginalis* (DNA level, ≥10^9^ copies/mL), had the highest predictive value for the diagnosis of BV [20]. As a strict anaerobe, it is not surprising that *A. vaginae* has not been previously recognized in women with BV. With culture-independent techniques, *A. vaginae* has been confirmed as an important component of the complex bacterial ecology that constitutes abnormal vaginal microbiota [21]. It was unexpected that the relative abundance of *Prevotella* in the BV-positive group was so high that *Prevotella* could be used for BV diagnosis alone or in combination with other vaginal pathogenic bacteria [22]. *Eggerthella*, like *A. vaginae*, was strongly correlated with BV in our study. In addition, Fredricks *et al.* (2005) showed that detection of *Leptotrichia*/*Sneathia* spp. was very specific for BV by bacterium-specific PCR assays [3]. Our observations also showed that the prevalence of *Leptotrichia*/*Sneathia* in the BV-positive group was higher than BV-negative subjects, which could be a new marker for BV in the pathogenic vaginal communities.

In the healthy condition, lactobacilli-dominated vaginal microbiota has been thought to play a major role in protecting the vaginal environment from non-indigenous and potentially harmful microorganisms. This is accomplished through the production of lactic acid, resulting in a low and protective pH (3.5–4.5). A high vaginal pH is indicative of changes within the vaginal microbiota and is used as a criterion in the diagnosis of BV [11]. Clarke *et al*. (2012) also reported that elevated vaginal pH is positively related to detection of HPV in a very large cohort of randomly selected women [23]. Vaginal pH reflects a combination of factors affecting the vaginal microenvironment. The present data demonstrated that there were negative associations between vaginal pH and *Lactobacillus*. Previous studies have also shown that the vaginal pH is strongly related to age and menopausal status [23]–[25]. As the relative lack of H_2_O_2_- and acid-producing *Lactobacillus* and overgrowth of anaerobic bacteria, such as *G. vaginalis*, *A. vaginae*, *Leptotrichia*/*Sneathia* spp., *Eggerthella* spp., *Megasphaera* spp. and *Prevotella* spp., the vaginal pH would increase dramatically and BV-associated signs and symptoms would subsequently occur. Research has shown that lactic acid is more effective than acidity alone as a microbicide against HIV or pathogens, such as *Neisseria gonorrhoeae* [26], [27]. These bacteria in the vaginal pathogenic community cannot produce lactic acid to lower the vaginal pH, which has a significantly positive relationship with elevated vaginal pH. For women of reproductive age, the composition of vaginal microbiota might be a key determinant for maintaining a low vaginal pH.

The changing patterns between vaginal pathogenic bacteria and Nugent scores were similar with vaginal pH. The Nugent scoring system for BV is based on the total number of large gram-positive rods (*Lactobacillus* morphotypes), the number of small gram-variable and gram-negative rods (*G vaginalis*, *Bacteroides*, and *Prevotella* morphotypes), and curved gram-negative rods. A score of 0–3 is representative of normal microbiota, a score of 4–6 is designated intermediate and corresponds to a disturbed or altered microbiota, and a score of 7–10 is consistent with BV microbiota. In the present study, only women with Nugent scores ≥7 were chosen for analysis. Consistent with the previous study conducted by Ravel *et al.* (2011), *Lactobacillus*-dominated vaginal microbiota were negatively correlated with Nugent scores [9], with the exception of *L. jensenii*. Those bacteria in the vaginal pathogenic community, most of which were gram-variable and gram-negative rods, were positively associated with Nugent scores [9]. Based on the present study, the vaginal pathogenic community might correspond to a cluster 3 of the vaginal microbiome in the Ravel's study [9]. Tamrakar *et al.* (2007) also reported that the presence of *Eggerthella* was an independent risk factor for BV scores (Nugent score ≥7) [28]. Thus, there was no doubt that vaginal pathogenic bacteria as a whole were significantly correlated with Nugent scores.

There were several limitations in our study. We realized that we did not have adequate information regarding different vaginal conditions such as intermediate Nugent scores (4–6) which could be useful in verifying the role of these bacteria in the vaginal pathogenic community. Secondly, a new cohort of women suffered from BV was needed to validate the diagnostic values of the bacteria in the vaginal pathogenic community for BV. Furthermore, the present study should have larger our sample sizes to elucidate the relationship between vaginal pathogenic community and vaginal pH and Nugent scores more clearly.

In conclusion, the vaginal pathogenic community including *Gardnerella*, *Atopobium*, *Eggerthella*, *Megasphaera* typeI, *Leptotrichia*/*Sneathia* and *Prevotella* can be used as targets for BV diagnosis. These bacteria in the vaginal pathogenic community were positively related with vaginal pH and Nugent scores. Our present study indicates the vaginal pathogenic community, rather than a single vaginal microorganism, was participated in the onset of BV directly.
